# Long intergenic non-protein coding RNA 02570 promotes nasopharyngeal carcinoma progression by adsorbing microRNA miR-4649-3p thereby upregulating both sterol regulatory element binding protein 1, and fatty acid synthase

**DOI:** 10.1080/21655979.2021.1979317

**Published:** 2021-09-21

**Authors:** Fei Liu, Jiazhang Wei, Yanrong Hao, Jiao Lan, Wei Li, Jingjin Weng, Min Li, Cheng Su, Bing Li, Mingzheng Mo, Fengzhu Tang, Yongli Wang, Yong Yang, Wei Jiao, Shenhong Qu

**Affiliations:** a Research Center of Medical Sciences, The People’s Hospital of Guangxi Zhuang Autonomous Region, Guangxi Academy of Medical Sciences, Nanning City, P.R. China; b Department of Otolaryngology & Head and Neck, The People’s Hospital of Guangxi Zhuang Autonomous Region, Guangxi Academy of Medical Sciences, Nanning City, P.R. China; c Cancer Center, The People’s Hospital of Guangxi Zhuang Autonomous Region, Guangxi Academy of Medical Sciences, Nanning City, P.R. China; d Health Management Center, The People’s Hospital of Guangxi Zhuang Autonomous Region, Guangxi Academy of Medical Sciences, Nanning City, P.R. China

**Keywords:** LINC02570, nasopharyngeal carcinoma (NPC), miR-4649-3p, SREBP1, FASN, progression

## Abstract

Our previous studies have elucidated a possible connection between long intergenic non-protein coding RNA 2570 (LINC02570) and nasopharyngeal carcinoma (NPC). However, the precise mechanism by which LINC02570 promotes NPC remains unknown. We used quantitative polymerase chain reaction (qPCR) to detect LINC02570 expression in nasopharyngeal cell lines, NPC tissues, and chronic rhinitis tissues. Subcellular LINC02570 localization was confirmed by fluorescence *in situ* hybridization (FISH). The effects of LINC02570 stable knockdown and overexpression on viabillity, proliferation, migration, and invasion were analyzed using 3-(4,5-Dimethyl-2-Thiazolyl)-2,5-Diphenyl-2-H-Tetrazolium bromide (MTT), a colorimetric focus-formation assay, a wound healing assay, and transwell assays. RNA crosstalk analysis *in silico* predicted microRNA-4649-3p (miR-4649-3p) binding to LINC02570 or sterol regulatory element binding transcription factor 1 (SREBF1). A dual luciferase reporter assay was used to confirm potential interactions. Sterol regulatory element binding protein 1 (SREBP1) and fatty acid synthase (FASN) expression were detected by western blotting. The results suggest that LINC02570 is upregulated in late clinical stage NPC patients, and promotes NPC progression by adsorbing miR-4649-3p to up-regulate SREBP1 and FASN. This study elucidates a potential chemotherapeutic target involved in lipid metabolism in NPC.

## Introduction

Nasopharyngeal carcinoma (NPC) occurs with a high incidence rate in East and Southeast Asia, especially in southern China, including Guangdong, Guangxi, and Hong Kong [1–3]. Some NPC patients are difficult to diagnose in early clinical stages, because they show no obvious symptoms. Unfortunately, NPC patients can suffer tremendous pain due to metastatic foci. High recurrence and metastasis are the main causes of poor prognosis and death in NPC patients [4]. Epstein-Barr virus (EBV) infects nasopharyngeal epithelial cells, and plays an important role in NPC progression [5]. Plasma EBV DNA is screened for in early stages of NPC by quantitative polymerase chain reaction (qPCR) and sequencing analysis [6,7]. Some candidate biomarkers, such as anti-EBV antibodies and microRNAs (miRNAs), are also detected in early stages of NPC [8]. Biomarkers have been shown to be useful for diagnosis, and for assessing NPC prognosis and progression [9–12].

Long noncoding RNAs (lncRNAs) comprise at least 200 nucleotides, and mediate important functions involved in regulating chromatin dynamics, gene expression, cell growth, and development [13]. Some putative lncRNAs encode small proteins or peptides [14]. Increasing evidence supports close associations between lncRNAs and NPC progression [15–17]. Mechanistically, lncRNAs mainly regulate target gene expression by adsorbing miRNAs. For instance, LINC00667 promotes NPC progression by adsorbing miR-4319, increasing levels of forkhead box Q1 (FOXQ1) [18]. MSC antisense RNA 1 (MSC-AS1) exacerbated NPC progression by adsorbing miR-524-5p to upregulate nuclear receptor subfamily 4 group A member 2 (NR4A2) [19].

In previous studies, we found that zinc finger and BTB domain containing 7A (ZBTB7A) has complex functions in NPC progression [20–23]. High expression of the proto-oncogene ZBTB7A enhanced the viability, migration, and invasion abilities of stably transfected NPC cells [23]. Nevertheless, tumorigenicity is maintained in stably transfected NPC cells expressing ZBTB7A at low levels [22]. To elucidate mechanisms by which ZBTB7A could maintain NPC tumorigenicity, 1501 differentially expressed lncRNAs and 1276 messenger RNAs (mRNAs) were identified by microarray-based screening comparing cells stably transfected with a short hairpin RNA (shRNA) targeting ZBTB7A with cells stably transfected with empty vector. We found that stable reduction of ZBTB7A upregulated some oncogenes through an lncRNA microarray [23]. Compensatory mechanisms may explain why a few late clinical stage NPC patients exhibited low ZBTB7A expression.

Some differentially expressed mRNAs and lncRNAs have been validated by qPCR in NPC and chronic rhinitis tissues. Two lncRNAs (ENST00000442852, NR_047538) and two mRNAs (ZBTB7A, fatty acid synthase [FASN]) display higher levels in NPC tissues than in control tissues. We also found that sterol regulatory element binding transcription factor 1 (SREBF1), the gene encoding sterol regulatory element binding protein 1 (SREBP1), was upregulated in cells with stable knockdown of ZBTB7A [23]. It is well known that FASN is upregulated by SREBP1. Both of these proteins are key factors in lipid metabolism [24]. NR_047538, which is downregulated in cells with stable knockdown of ZBTB7A, promotes NPC progression [25]. By contrast, the function of ENST00000442852 (gene symbol: XXbac-BPG27H4.8), also known as long intergenic non-protein coding RNA 2570 (LINC02570) in NPC remains unknown. Based on increased LINC02570 expression in NPC relative to control cells [23], we hypothesized that it may play a positive role in NPC progression. To validate this hypothesis, we explored possible mechanisms of LINC02570 in NPC through *in vitro* studies and clinical and bioinformatic analyses.

## Materials and methods

### Tissue samples

These studies were approved by the Ethics Committee of the People’s Hospital of Guangxi Zhuang Autonomous Region. Sixty NPC and 20 chronic rhinitis tissues were acquired [23]. To confirm a functional relationship between miR-4649-3p and NPC, 20 additional NPC and chronic rhinitis tissues each were collected from March 2019 to July 2019. Tumors were staged based on the Union for International Cancer Control (UICC, 2010, 7^th^ edition) staging system for NPC. All specimens were collected from patients prior to therapy. Informed consent was obtained from all patients before the study.

### Bioinformatic analyses

Binding sequences within LINC02570, unknown miRNAs, and SREBF1 were analyzed using TargetScan (http://www.targetscan.org/vert_72/) and DIANA (http://carolina.imis.athena-innovation.gr/diana_tools/web/index.php) [26]. These analyses indicated that miR-4649-3p binds to LINC02570 and SREBF1. Coincidentally, miR-4649-3p suppresses NPC proliferation [27].

### Cell culture

NPC cell lines HK1, C666-1, SUNE-1, 5–8 F, 6–10B, and CNE3 were cultured in Roswell Park Memorial Institute 1640 medium supplemented with 10% fetal bovine serum (Gibco, Thermo Fisher Scientific, Waltham, USA). The immortalized nasopharyngeal epithelial cell line NP69 was cultured in keratinocyte-serum-free medium with 5% bovine pituitary extract and recombinant epidermal growth factor (Gibco) [22,23].

### Cell transfection

Lentiviruses overexpressing (OE) or knocking down (KD-1#, KD-2#, and KD-3#) LINC02570 were purchased from Shanghai Research & Science Biotechnology (Shanghai, China). Lentivirus empty vectors were used as negative controls (NCs). NPC cell lines were seeded in 6-well plates and grown to 50% confluence. Transfections were performed according to the manufacturer’s instructions. Stably transfected cells were selected for using blasticidin (Solarbio, Beijing, China), continuously passaged, and checked by qPCR. After 10 passages, we considered sublines to be stably transfected with the lentiviruses. The miR-4649-3p mimic, inhibitor, and NC were purchased from Guangzhou RiboBio (Guangzhou, China), and were transiently transfected into NPC cells using Lipofectamine 3000 (Invitrogen, Carlsbad, USA) [27].

### qPCR

Total RNA was extracted from tissues and cells using TRIzol reagent (Invitrogen). Reverse transcription and qPCR were performed on RNA from 80 tissues [23], to assess expression of genes including SREBF1 (Supplementary Materials, Figure S1). ZBTB7A expression correlated negatively with SREBF1 expression (Supplementary Materials, Figure S2). Total RNA from cell lines was reverse transcribed using a RevertAid First Strand cDNA Synthesis Kit (Fermentas, Thermo Fisher Scientific). miRNAs from the cell lines and 40 additional tissues were extracted using a miRcute miRNA Isolation Kit (Tiangen, Beijing, China), and reverse transcribed using a miRcute Plus miRNA First-Strand cDNA Kit (Tiangen). qPCR reactions were performed using Fast Start Universal SYBR Green Master mix (Roche, Basel, Switzerland) or a miRcute Plus miRNA qPCR Kit (Tiangen) in a 7500 real-time PCR system (Applied Biosystems, Thermo Fisher Scientific). Relative RNA expression was calculated using the 2^−ΔΔCt^ method, and normalized to human beta actin (hACTB) or U6 [28]. The following primers were used for qPCR reactions: LINC02570, forward: 5ʹ-TGTGAGTACGCCTGGCTTTT-3ʹ, reverse: 5ʹ-CCTTTCTCCTGGTCATTTGTTC-3ʹ; SREBF1, forward: 5ʹ-GCTGTTGGTGCTCGTCTCCTTG-3ʹ, reverse: 5ʹ-GCTTGCGATGCCTCCAGAAGTAC-3ʹ; FASN, forward: 5ʹ-TCCGAGTCTCCTGACCACTACCT-3ʹ, reverse: 5ʹ-GCAGCACCACATCCTCAAACA-3ʹ; hACTB, forward: 5ʹ-GCACCCAGCACAATGAAGA-3ʹ, reverse: 5ʹ-AATAAAGCCATGCCAATCTCA-3ʹ; miR-4649-3p: forward: 5ʹ-ACACTCCAGCTGGGTCTGAGGCCTGCCTC-3ʹ, reverse: 5ʹ-CTCAACTGGTGTCGTGGAGTCGGCAATTCAGTTGAGTGGGGA-3ʹ; U6, forward: 5ʹ-CTTCGGCAGCACATATAC-3ʹ, reverse: 5ʹ-GGCCATGCTAATCTTCTC-3ʹ.

### RNA fluorescence in situ hybridization (FISH) assay

Cy3-labeled LINC02570 probe was acquired from Guangzhou RiboBio. Subcellular localization of LINC02570 was investigated using a FISH Kit (RiboBio). NPC cells were seeded onto cell culture slides in 24-well plates at a density of 5 × 10^4^ cells/well and grown to 60% confluence. Cells were fixed in 4% paraformaldehyde for 10 min at room temperature, prehybridized for 30 min at 37°C after permeabilization, then hybridized overnight at 37°C with a mixture of 100 μL hybridization solution and 2.5 μL 20 μM Cy3-labeled LINC02570 probe. Cell nuclei were stained with 4,6-diamidino-2-phenylindole (DAPI) for 10 min at room temperature. Fluorescent images were acquired using a TCS SP8 laser scanning confocal microscope (Leica, Wetzlar, Germany) [29].

### 3-(4,5-Dimethyl-2-Thiazolyl)-2,5-Diphenyl-2-H-Tetrazolium Bromide (MTT) assay

Cell sublines were seeded into 96-well plates at a density of 1500 cells/well, and incubated with 0.5% MTT (Sigma-Aldrich, Saint Louis, USA) for 4 h. Cells were then lysed with dimethyl sulfoxide (DMSO, Sigma-Aldrich) and absorbance was measured using a Synergy H1 microplate reader (BioTek Instruments, Winooski, USA) [22].

### Colorimetric focus-formation assay

Cell sublines were seeded into 6-well plates at a density of 100 cells/well and fixed with 100% methanol after 2 weeks. They were then stained with 5% crystal violet (Amresco, Houston, USA) in 100% methanol [22].

### Wound healing assay

To exclude cell proliferation effects, cell sublines were incubated in serum-free medium for 24 h before assay. They were then seeded into 6-well plates at a density of 10^6^ cells/well. Gaps were made by manually scraping cell monolayers with a 200 μL sterile pipette tip. Cell migration was observed at 0 and 24 h. Five microscopic fields were randomly selected and analyzed. Images were acquired using an IX71 fluorescence microscope (Olympus, Tokyo, Japan). Cell-covered areas within scrape zones were measured at different time points [22].

### Transwell migration and invasion assays

Cell sublines were incubated in serum-free medium for 24 h, then seeded into 6.5 mm transwell chambers in 24-well plates (pore size: 8 μm, Costar, Corning, NY 14831, USA) at a density of 5 × 10^4^ cells/well (migration) or 10^5^ cells/well (invasion). For invasion assays, the upper chamber surface was covered with a mixture of 17.5 μL Matrigel basement membrane matrix (BD Biosciences, San Jose, USA) and 52.5 μL serum-free 1640 medium. Non-migratory and noninvasive cells on the upper surface of the filters were completely removed using cotton swabs after 24 h. Migratory and invasive cells on the lower surface of the filters were washed with phosphate buffered saline (PBS), fixed with methanol, and stained with 1% crystal violet. Migratory and invasive cells were quantitated using a BX51 microscope (Olympus) [22].

### Western blotting

Cells were lysed using radioimmunoprecipitation buffer (Beyotime, Shanghai, China). Total protein (20 μg) was separated by 8% sodium dodecyl sulfate-polyacrylamide gel electrophoresis and transferred onto 0.45 μm polyvinylidene fluoride membranes (Millipore, Billerica, USA) using a Mini-Protean System (Bio-Rad, Hercules, USA). Membranes were incubated with primary antibody (1:500 dilution, catalog no. ABS1508, anti-SREBP1 antibody from Sigma-Aldrich; 1:500 dilution, catalog no. 3189S, anti-FASN antibody from Cell Signaling, Danvers, USA; 1:1 200 dilution, catalog no. AA128, anti-ACTB antibody from Beyotime) overnight at 4°C, then incubated with horseradish peroxidase-conjugated anti-rabbit antibody from Beyotime (1:8 000 dilution, catalog no. A0208) or anti-mouse antibody from Beyotime (1:12,000 dilution, catalog no. A0216) for 2 h at 37°C. Enhanced chemiluminescence (Beyotime) was added to the membranes, which were detected using an Odyssey Fc Imaging System (Li-Cor, Lincoln, USA) [22].

### Dual Luciferase Reporter Assay

LINC02570 wild-type (WT)/mutant (Mut) and SREBF1 WT/Mut were co-transfected with miR-4649-3p mimic and mimic NC into NPC cells. Relative luciferase activity was detected using a Dual-Luciferase® Reporter Assay System (Promega, Madison, USA) according to the manufacturer’s instructions [27].

### Statistical analyses

All assays were executed independently in triplicate. All data were calculated as means ± standard deviation (SD), except for the median from the unregular distribution of LINC02570 in NPC tissues. Two-tailed Student’s *t*-tests were used to compare two independent groups. One-way ANOVA was used to compare multiple groups. Otherwise, unpaired Student’s *t*-test or Fisher’s exact test was used for statistical analyses appropriate to experimental conditions. The Mann Whitney test was used to assess scatter plots of NPC tissues analyzed by qPCR if variances based on the F test were significantly different. Kaplan–Meier survival curves were used for univariate survival analyses. The log-rank test was performed to compare survival curves between two groups. In these studies, statistical significance was set at *p* < 0.05 [23].

## Results

### LINC02570 is upregulated in late clinical stage NPC patients

**Figure 1.**
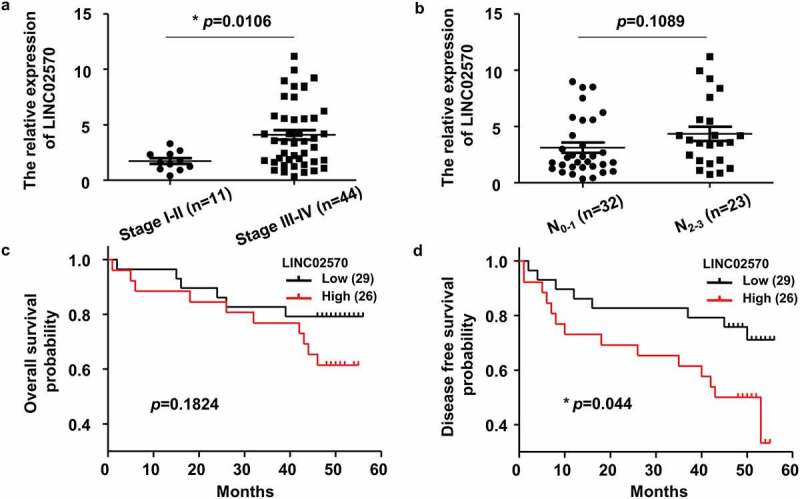
LINC02570 is upregulated in late clinical stage NPC patients. (a) LINC02570 expression in NPC patients in early or late clinical stages. *p < 0.05. (b) LINC02570 expression in NPC patients with non-advanced or advanced lymph node metastatic disease. (c) Kaplan-Meier analysis of overall survival (OS) among NPC patients with high or low LINC02570 expression. (d) Kaplan-Meier analysis of disease-free survival (DFS) among NPC patients with high or low LINC02570 expression. *p < 0.05

To explore the role of LINC02570 in NPC progression, its mRNA levels were detected by qPCR in control and NPC tissues, and survival data for NPC patients were analyzed. In a previous study, LINC02570 expression in 60 NPC tissues was higher than that in 20 chronic rhinitis tissues [23]. Fifty-five NPC patients were followed up after definite diagnoses in our hospital, while five patients were not. LINC02570 expression was higher in late-stage (III–IV) than in early stage (I–II) NPC (Figure 1(a)). However, there was no obvious difference between elementary N stage (N0-1) and advanced N stage (N 2–3) NPC (Figure 1(b)). The 55 patients were assigned to high (n = 26, LINC02570 > 2.5) and low (n = 29, LINC02570 ≤ 2.5) expression groups. The median of all LINC02570 results was 2.5. Obvious differences in overall survival (OS) between groups were not observed (Figure 1(c)). However, patients with high LINC02570 expression showed poorer prognosis for disease-free survival (DFS) than those with low expression (Figure 1(d)).

### Stable knockdown/overexpression and location of *LINC02570* in NPC cell lines

**Figure 2.**
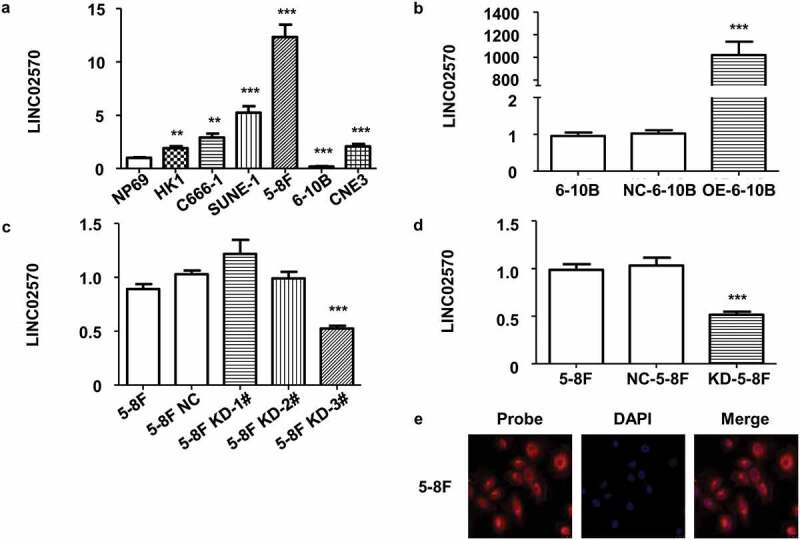
Stable overexpression/knockdown, and LINC02570 location in NPC cell lines. (a) LINC02570 expression in NPC cell lines and a nasopharyngeal epithelial cell line. NP69 was a noncancerous control for NPC cell lines. (b) LINC02570 expression was stably up-regulated. 6–10B and NC-6-10B were controls relative to OE-6-10B. (c) LINC02570 was transiently knocked down in 5–8 F. 5–8 F and 5–8 F NC were considered control samples for 5–8 F KD-1#, 2# and 3#. (d) LINC02570 was stably knocked down. 5–8 F and NC-5-8 F were control samples for KD-5-8 F. (e) LINC02570 was mainly located in cytoplasm (magnification: 400x). **p < 0.01, ***p < 0.001

To explore the association between changes in LINC02570 expression and NPC progression, cell sublines with stable knockdown or overexpression of LNC02570 were constructed by lentiviral transfection and selection for stable transformants. The location of LINC02570 in NPC cells was detected by FISH assay. LINC02570 expression was higher in the 5 NPC cell lines than in the NP69 nasopharyngeal epithelial cell line (Figure 2(a)). Two NPC cell lines were chosen and transfected using lentiviral constructs for knockdown or overexpression. The chosen cell lines displayed the highest (5–8 F) or lowest (6–10B) LINC02570 expression. These cell lines were transiently transfected with the lentiviruses. Stably transfected cell lines were then selected for over 10 passages. LINC02570 expression was effectively elevated or decreased in different sublines stably transfected with lentiviruses (Figure 2(b), 2(c) and 2(d)). They were named OE-6-10B and KD-5-8 F, respectively.

Sublines with lentiviral empty vectors were used as negative controls, and named NC-6-10B and NC-5-8 F. To detect LINC02570 subcellular localization in NPC cells, 5–8 F was selected for FISH assay due to its highest expression of LINC02570 among the NPC cell lines. The amount of LINC02570 in the cytoplasm was obviously higher than that in the nucleus. The results showed that LINC02570 is mainly located in the cytoplasm (Figure 2(e)).

### Stable knockdown of LINC02570 suppressed NPC progression

**Figure 3.**
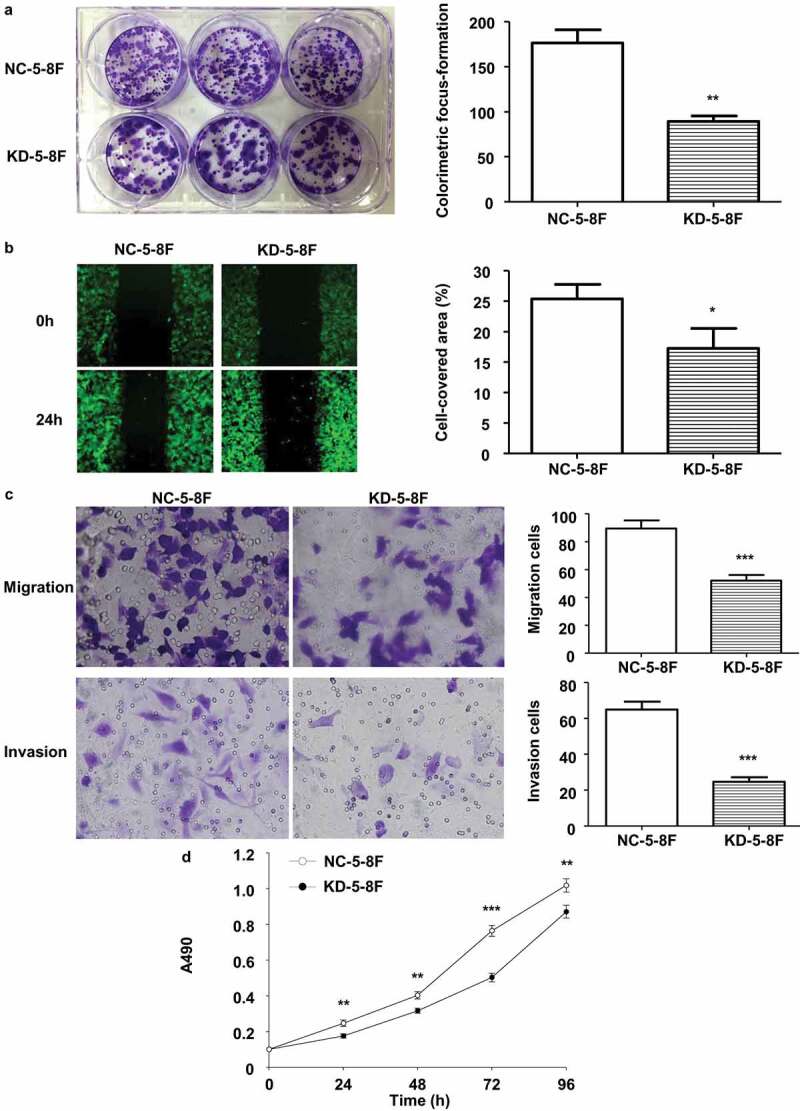
Stable LINC02570 knockdown attenuated proliferation, migration, invasion and viability of NPC cells. (a) Proliferation of NC-5-8 F and KD-5-8 F cells was detected by colorimetric focus-formation assay. (b) Migration of NC-5-8 F and KD-5-8 F cells was detected by wound healing assay (magnification: 40x). Stably transfected cells displayed green fluorescence. (c) Migration and invasion of NC-5-8 F and KD-5-8 F were detected by transwell assays (magnification: 200x). (d) NC-5-8 F and KD-5-8 F cell viability was detected by MTT. *p < 0.05, **p < 0.01, ***p < 0.001

To explore the association between stably low LINC02570 expression and NPC progression, the oncogenic characteristics of NPC cells were assayed *in vitro*. By colorimetric focus-formation, wound healing, transwell assays, and MTT assays, proliferation, migration, invasion, and viability of KD-5-8 F were weaker than those of NC-5-8 F (Figure 3).

### Stable *LINC02570* overexpression promotes NPC progression

**Figure 4.**
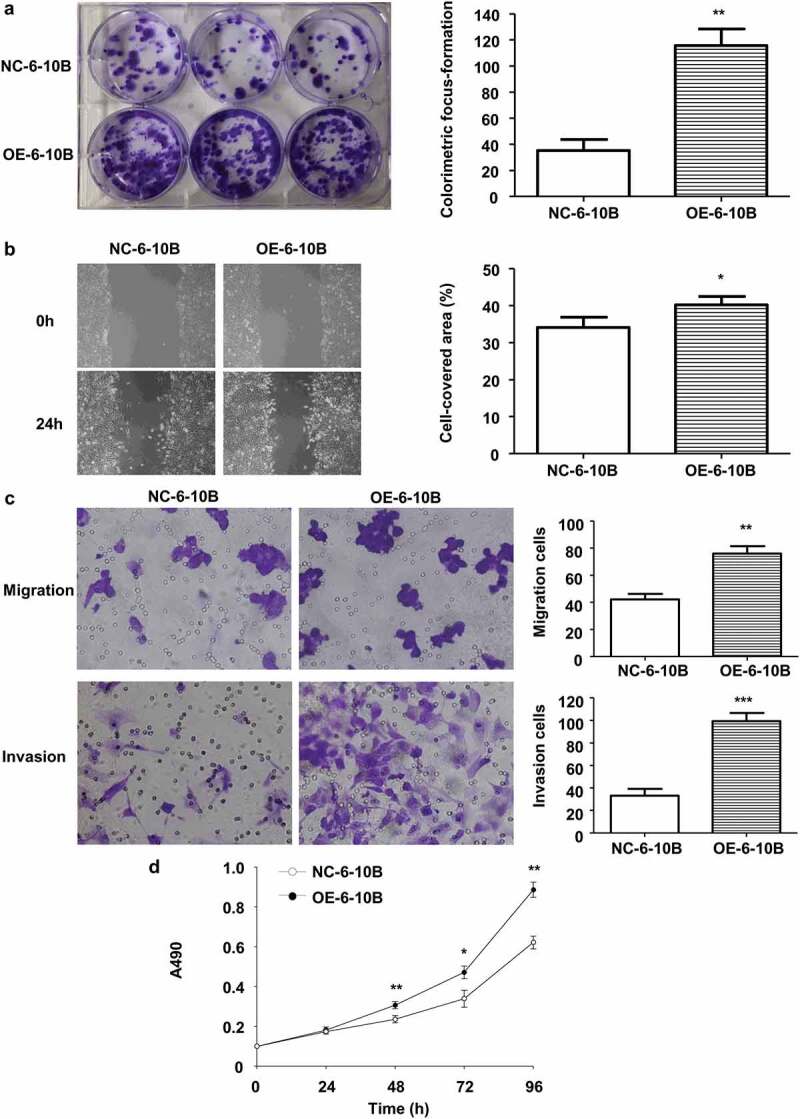
Stable LINC02570 overexpression enhanced NPC cell proliferation, migration, invasion and viability. (a) Proliferation of NC-6-10B and OE-6-10B cells was detected by colorimetric focus-formation assay. (b) Migration of NC-6-10B and OE-6-10B cells was detected by wound healing assay (magnification: 40x). Stably transfected cells didn’t show any fluorescence. (c) NC-6-10B and OE-6-10B cell migration and invasion were detected by transwell assays (magnification: 200x). (d) NC-6-10B and OE-6-10B cell viability was detected by MTT. *p < 0.05, **p < 0.01, ***p < 0.001

To explore the association between stable high LINC02570 expression and NPC progression, the oncogenic characteristics of NPC cells were assessed *in vitro*. Proliferation, migration, invasion, and viability of OE-6-10B cells were stronger than those of NC-6-10B (Figure 4).

#### LINC02570 regulates the SREBP1-FASN signaling pathway in NPC

**Figure 5.**
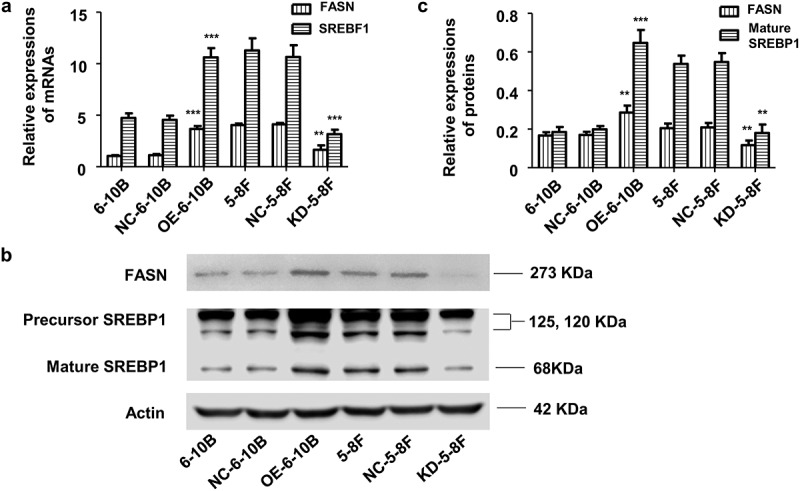
SREBP1 and FASN expression in stably transfected 6–10B and 5–8 F cells. (a) SREBF1 and FASN mRNA levels were detected by qPCR. (b) and (c) SREBP1 and FASN protein levels were detected by western-blot. SREBF1 refers to the transcript encoding SREBP1. 6–10B and NC-6-10B were control samples for OE-6-10B. 5–8 F and NC-5-8 F were control samples for KD-5-8 F. **p < 0.01, ***p < 0.001

To explore the possible mechanism by which LINC02570 regulates NPC progression, we assessed expression of important genes involved in lipid metabolism *in vitro*. SREBP1 and FASN expression was lower in KD-5-8 F than in NC-5-8 F according to qPCR and western blotting. However, SREBP1 and FASN expression was higher in OE-6-10B than in NC-6-10B (Figure 5).

*LINC02570 promotes NPC cell progression by adsorbing miR-4649-3p* to upregulate *SREBP1-FASN*

**Figure 6.**
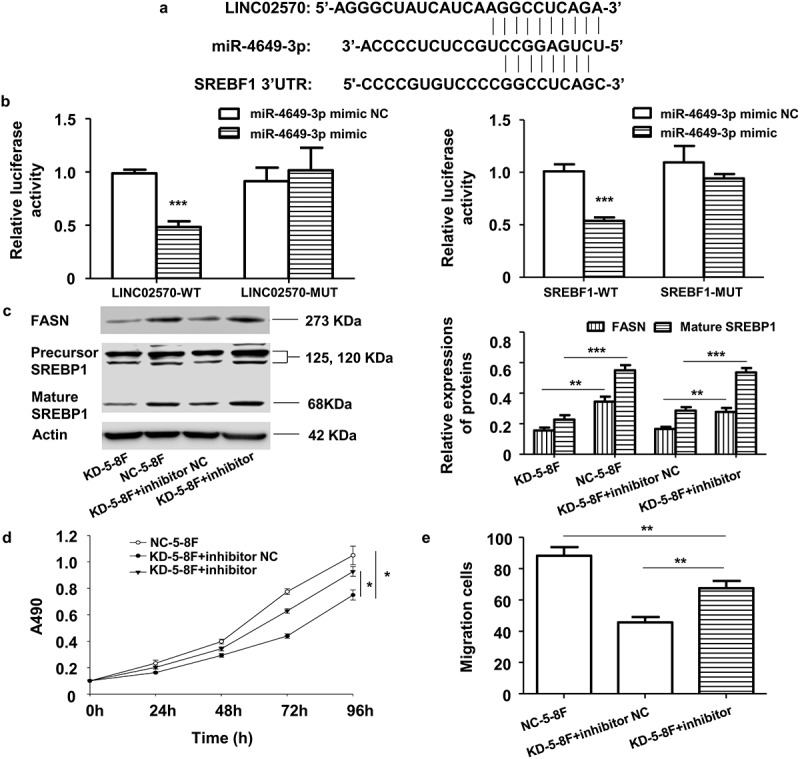
LINC02570 promotes NPC progression through the miR-4649-3p/SREBP1/FASN axis. (a) Bioinformatics analysis predicted miR-4649-3p binding sites on LINC02570 and SREBF1. (b) Luciferase activities of 5–8 F cells co-transfected with LINC02570 wild-type (WT)/mutant (Mut) or SREBF1 WT/Mut +miR-4649-3p mimic or mimic NC were respectively analyzed by dual luciferase reporter assay. (c) SREBP1 and FASN protein levels were detected by western blotting. (d) and (e) Viability and migration were detected by MTT and transwell assays. *p < 0.05, **p < 0.01, ***p < 0.001

To explore the specific mechanism by which LINC02570 regulates NPC progression via lipid biosynthesis pathways, connections between LINC02570 and some miRNAs were predicted and confirmed by bioinformatics and dual luciferase reporter assays. Interaction between LINC02570 and miR-4649-3p was confirmed *in vitro*. Bioinformatics analysis revealed that miR-4649-3p potentially bound to LINC02570 and SREBF1 (Figure 6(a)). Moreover, LINC02570-WT significantly inhibited luciferase activity in 5–8 F expressing the miR-4649-3p mimic (Figure 6(b)). miR-4649-3p obviously inhibited luciferase activity in 5–8 F expressing the SREBF1-WT plasmid (Figure 6 (c)). Furthermore, LINC02570-WT promoted SREBP1 and FASN expression by adsorbing miR-4649-3p (Figure 6(d) and (e)). As a result, viability and migration of KD-5-8 F were enhanced by inhibiting miR-4649-3p (Figure 6 (f) and (g)).

## Discussion

lncRNAs play complex roles in NPC. Some of them promote NPC progression [30,31], while others suppress NPC [32,33]. Because of the functional correlation between LINC02570 and NPC [23], we explored potential regulatory mechanisms. We have found through loss and gain of function assays that high LINC02570 expression obviously enhances NPC cell viability, proliferation, migration, and invasion. Moreover, elevated LINC02570 expression upregulated SREBP1 and FASN expression. Both are oncogenes in NPC [34]. Immediate early response 3 (IER3) is an mRNA encoding near neighbor of LINC02570 [23]. However, no obvious differences were observed between OE-6-10B and NC-6-10B, and negative result appeared between KD-5-8 F and NC-5-8 F (Supplementary Materials, Figure S3).

5–8 F and 6–10B are sublines of the NPC cell line SUNE-1. The tumorigenic and metastatic potentials of 5–8 F were stronger than those of 6–10B [35]. However, the invasiveness of OE-6-10B was stronger than that of NC-5-8 F. We found that SREBP1 and FASN protein levels were higher in OE-6-10B than in NC-5-8 F, indicating that a lipid biosynthesis pathway may promote NPC progression by enhancing LINC02570 expression.

miR-4649-3p suppresses NPC cell proliferation by binding to SHP-1, which is a SH2 domain containing protein tyrosine phosphatase [27]. We also confirmed that the relative expression of miR-4649-3p in NP69 cells was higher than that in NPC cell lines (Supplementary Materials, Figure S4). Furthermore, miR-4649-3p expression in rhinitis was higher than that in NPC tissues (Supplementary Materials, Figure S5). miR-4649-3p plays a suppressive role in NPC progression, acting as a bridge between LINC02570 and SREBP1/FASN. LINC02570 upregulated SREBP1/FASN expression and promoted NPC progression by absorbing miR-4649-3p.

## Conclusion

LINC02570 promotes NPC progression by modulating the miR-4649-3p/SREBP1/FASN axis. This study provides a potential chemotherapeutic target for lipid metabolism.

## Supplementary Material

Supplemental Material

## Article highlights

1. LINC02570 is frequently overexpressed in late stage NPC, and is associated with poor prognosis.
2. LINC02570 promotes NPC cell progression.
3. LINC02570 adsorbs and sequesters miR-4649-3p.
4. LINC02570 regulates the miR-4649-3p/SREBP1/FASN axis.
