# Supplementary figures and images for: Long intergenic non-protein coding RNA 02570 promotes nasopharyngeal carcinoma progression by adsorbing microRNA miR-4649-3p thereby upregulating both sterol regulatory element binding protein 1, and fatty acid synthase
Source: Bioengineered. 2021 Sep 21;12(1):7108–19. doi: 10.1080/21655979.2021.1979317 (PMC8806647; doi:10.1080/21655979.2021.1979317)

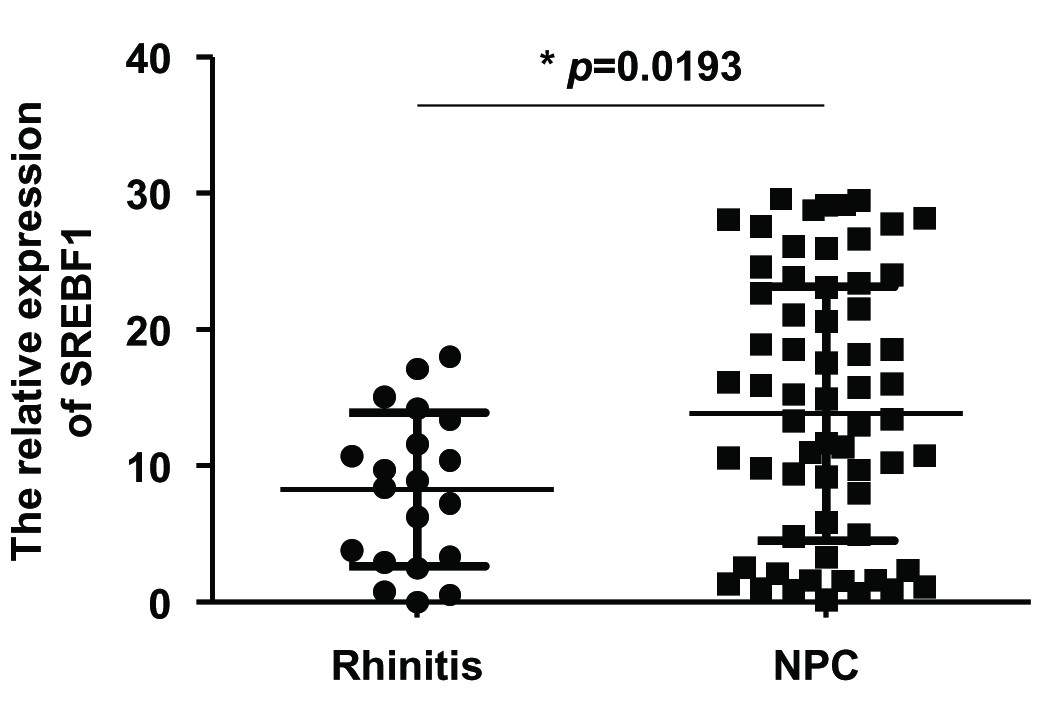

Supplement: Supplemental Material [file KBIE_A_1979317_SM7947.zip › Supplementary material/Figure S1 Validation of SREBF1 by qPCR in NPC and chronic rhinitis tissues.tif]

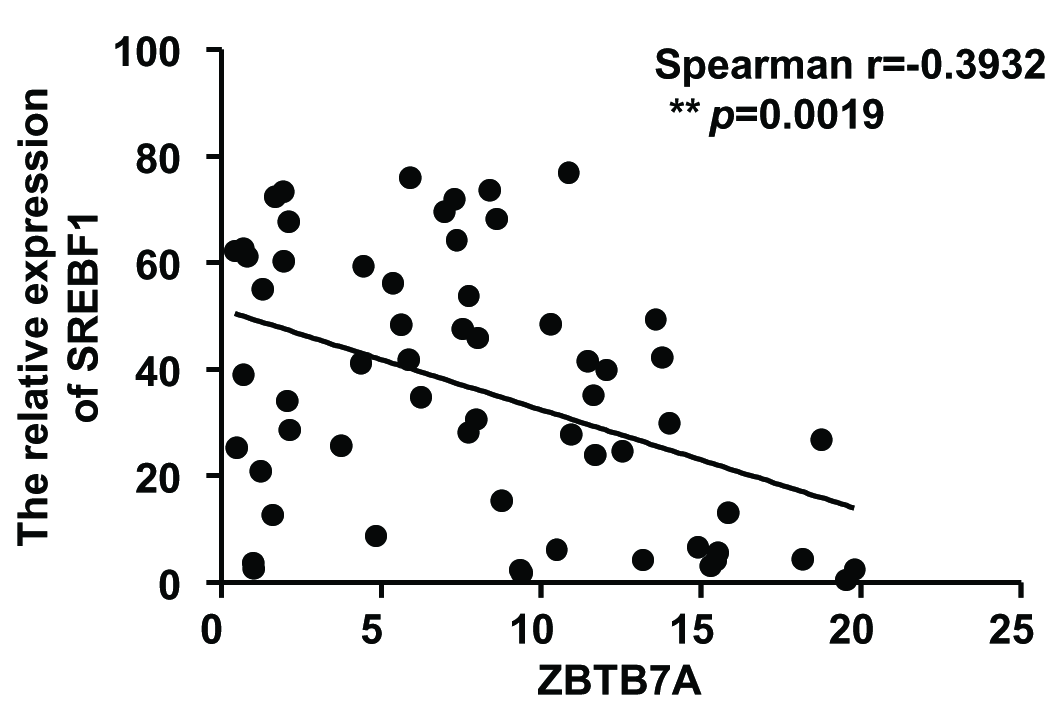

Supplement: Supplemental Material [file KBIE_A_1979317_SM7947.zip › Supplementary material/Figure S2 The association between ZBTB7A and SREBF1.tif]

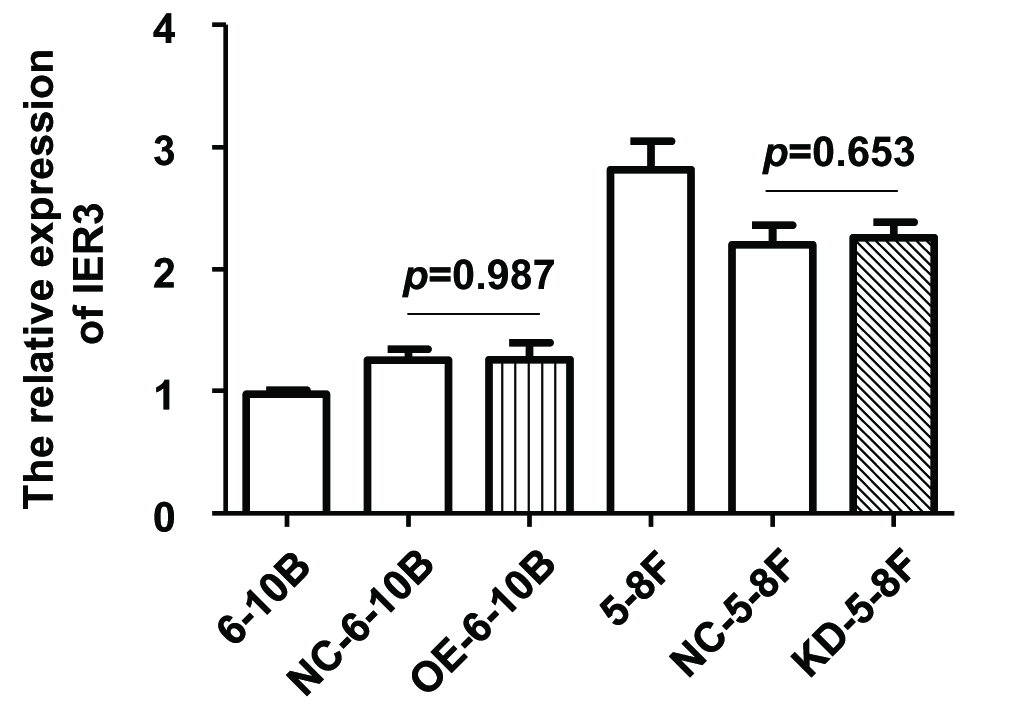

Supplement: Supplemental Material [file KBIE_A_1979317_SM7947.zip › Supplementary material/Figure S3 The mRNA expressions of IER3 in 6-10B and 5-8F stably transfected cells.tif]

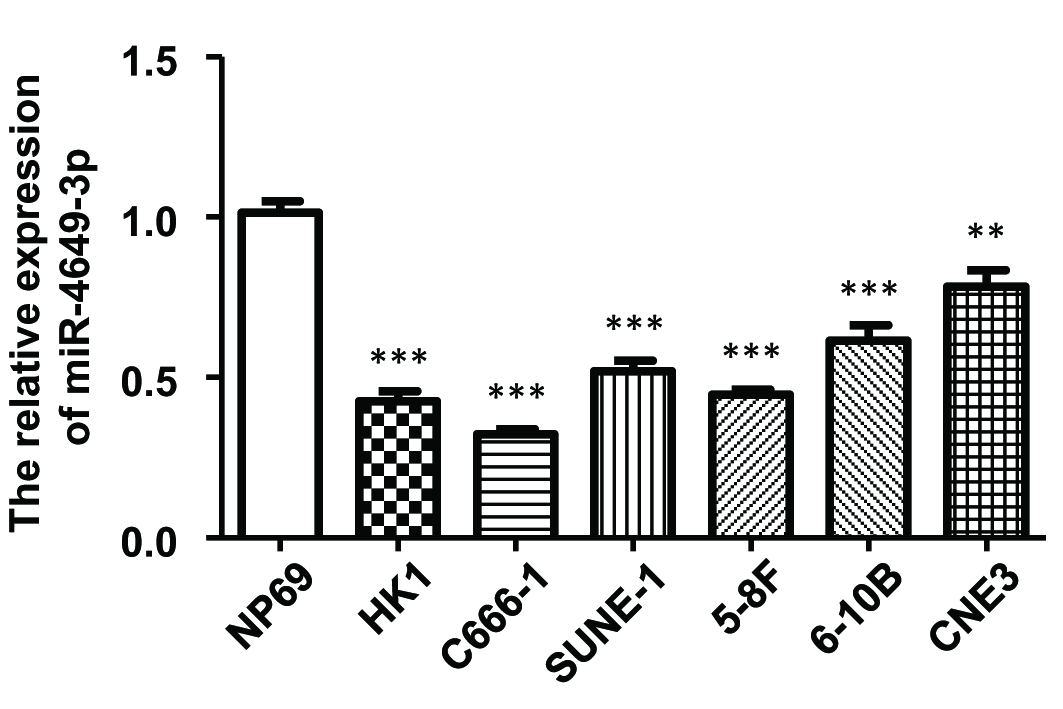

Supplement: Supplemental Material [file KBIE_A_1979317_SM7947.zip › Supplementary material/Figure S4 The relative expressions of miR-4649-3p in nasopharyngeal cell lines.tif]

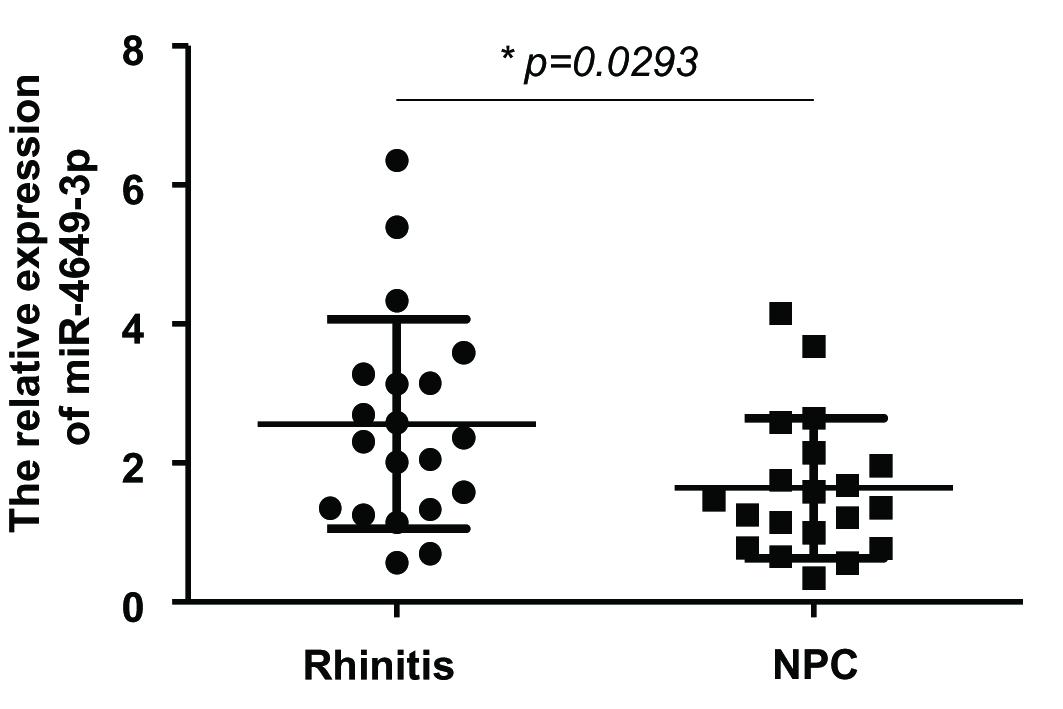

Supplement: Supplemental Material [file KBIE_A_1979317_SM7947.zip › Supplementary material/Figure S5 The expressions of miR-4649-3p in rhinitis and NPC tissues.tif]
